# Effects of Ciliate Infection on the Activities of Two Antioxidant Enzymes (SOD and CAT) in Captive Coral (*Goniopora columna*) and Evaluation of Drug Therapy

**DOI:** 10.3390/biology10111216

**Published:** 2021-11-21

**Authors:** Chiu-Min Cheng, Yu-Rong Cheng, De-Sing Ding, Ya-Ting Chen, Wei-Ting Sun, Chih-Hung Pan

**Affiliations:** 1 Department and Graduate Institute of Aquaculture, National Kaohsiung University of Science and Technology, Kaohsiung 811, Taiwan; cmcheng@nkust.edu.tw (C.-M.C.); 1051537103@nkust.edu.tw (W.-T.S.); chpan@nkust.edu.tw (C.-H.P.); 2 Department of Fisheries Production and Management, National Kaohsiung University of Science and Technology, Kaohsiung 811, Taiwan; yrcheng@nkust.edu.tw; 3 Department of Seafood Science, National Kaohsiung University of Science and Technology, Kaohsiung 811, Taiwan; melodyyu.chen@gmail.com

**Keywords:** ciliate, *Goniopora columna*, antioxidant enzymes, KCl, H_2_O_2_

## Abstract

**Simple Summary:**

In recent years, corals have been subjected to severe parasitic ciliates that have led to coral ulceration, bleaching or death. No previous studies have shown a treatment for ciliate infection of coral. In this study, two antioxidant enzymes (SOD and CAT) were used to judge the stress response in *Goniopora columna* after infection, and KCl and H_2_O_2_ were used to evaluate the therapeutic effect. According to the results, KCl 1.5% can effectively treat ciliate parasitism. This research has been successfully applied to the TCK Coral Farm to achieve effective treatment. It is very important for large-scale coral aquaculture.

**Abstract:**

Ciliate infection is a serious parasitic disease of coral. Infected coral rots and dies in a short time. In addition to killing corals by infecting them in the oceans, ciliate infection also poses a threat to corals farmed on a large scale. In this study, two antioxidant enzymes (SOD and CAT) were used to judge the stress response in *Goniopora columna* after infection, and KCl and H_2_O_2_ were used to evaluate the therapeutic effect. The results showed that SOD and CAT increased during the early stage of infection but decreased with the extension of infection time. In terms of drug therapy, it was found that the treatment of ciliate infection with 1.5% of KCl had no significant effect on SOD and CAT of *G. columna*. The morphological changes of zooxanthellae, chlorophyll a, and coral were not significant. H_2_O_2_ leads to a stress response and polyp contraction. In conclusion, 1.5% of KCl can be used in the selection of drugs to treat ciliate infection.

## 1. Introduction

Although there is little research on coral diseases, the damage caused by coral diseases is increasing. Studies have found that viruses, fungi, protozoa, ciliates, etc., may infect corals [1,2]. Ciliate disease is a recent coral disease that has attracted attention. It has been found that infected coral tissue can rot and die [1]. Previous studies have found that corals infected with folliculinid ciliate (*Halofolliculina corallasia*) develop a speckled black band in their tissues [3]. Corals have been found (*Acropora palmata*, *A. cervicornis* and *A. prolifera*) to be infected with *Halofolliculina corallasia* [4]. Ciliate infection has seriously affected coral health in coral reef areas in recent years [4]. Recent studies have found that ciliates infect corals with diseases; White Plague, White Band Disease (WBD) in the Caribbean, another Brb-like syndrome, and Brown Jelly syndrome are all caused by ciliate infections [2,5]. Verde et al. [6] found that WBD seriously affected the coverage of coral reefs in the Caribbean. Tissue regeneration was at least fifteen times slower than the mortality rates for ciliate diseases, regardless of coral species. Skeletal eroding band (SEB), which manifests as dense aggregations of the ciliate *H. corallasia*, was the first coral disease described from the Indo-Pacific [7]. A survey of Scleractinians and hydrocorals in the great barrier reef shows 2% of corals are infected with SEB [8]. Previous studies have found that brown band (BB), skeletal eroding band (SEB) and Caribbean ciliate infections (CCI) are have a wider geographical distribution, affecting important coral reef areas such as the Indo-Pacific, the Caribbean and the Great Barrier Reef [4,8]. According to observations [1], the parasite *Philaster lucinda* can be found in most infected coral tissues, therefore *P. lucinda* infection is a great threat to the survival of corals. At present, there are few studies on the infection and pathology of coral diseases caused by ciliates. *P. lucinda* is known to be parasitic in coral tissues, feeding on coral tissues and zooxanthellae, and entering and exiting the coral body cavity through the coral mouth [1]. At Taiwan Coral King (TCK) Coral Farm (Pingtung, Taiwan), we also found that *P. lucinda* infects *Goniopora columna*, *Euphyllia glabrescens*, and *Briareum violacea* in cultured corals. Coral tissue infection can fester, produce yellow or brown jellylike organizations, which have been found consist mostly of ciliate. Microscopic observations have found that ciliates have many zooxanthellae inside their bodies, yet there is no study outlining whether ciliates digest zooxanthellae as a form of nutrition; however, it is known that ciliate infection causes the breeding of canker, bleaching, and death.

The biological metabolism requires oxygen, and organisms produce reactive oxygen species (ROS) and free radicals. When corals are stressed, ROS production increases, leading to bleaching and even death [9,10]. Environmental and disease infection stress can induce the increased production of reactive oxygen species (ROS), leading to significant oxidative damage to a coral–algae symbiotic system [11,12,13,14]. When corals are stressed, they produce antioxidant enzymes to suppress ROS. The cellular response to the formation of oxygen radicals includes many defense mechanisms [15]. For example, enzymes such as superoxide dismutase (SOD) and catalase (CAT) act in concert to inactivate superoxide radicals (•O2^−^) and hydrogen peroxide (H_2_O_2_). This prevents the formation of the most reactive form of ROS, the hydroxyl radical (•OH), and subsequent cellular damage [14]. SOD catalyzes the dismutation of superoxide into oxygen and H_2_O_2_, and CAT is responsible for inactivating H_2_O_2_ into water and oxygen. These enzymes are responsible for detoxifying ROS, and their elevated activities indirectly indicate an increased production of ROS in corals as a result of disease or environmental stresses such as disease infection, temperature, irradiance, and UV radiation [11]. In this study, the changes in superoxide dismutase (SOD) and catalase (CAT) in coral were detected after ciliate infection to judge the stress response of ciliate infection to coral cells.

Coral bleaching or death occurs when the stress response caused by ciliate infection causes corals to become overloaded [10]. According to current studies, increased reactive oxygen species (ROS) is the cause of coral bleaching [16]. Outbreaks of disease and infection during captivity can cause mass coral deaths. At present, there have been no experiments on coral infection by ciliates to determine the damage caused to coral after infection. This has led to the widespread belief that these ciliates are opportunistic, eating dead and dying coral tissue caused by another unknown pathogen [1]. Large-scale coral aquaculture or aquarium will keep different varieties of coral in the same tank at the same time; if ciliate infection occurs, it will lead to large-scale infection in a short time, resulting in rapid decay and death of coral in a short time. Therefore, ciliate infection has a great impact on large-scale coral aquaculture and aquarium. However, studying the specific effects of ciliates on coral and treatment methods will be of great help to the large-scale coral aquaculture.

In this study, ciliates were purified and cultured from *G*. *columna* tissues, and were then used to infect corals, so as to observe the changes in coral morphology, zooxanthellae, chlorophyll a, and mortality and to detect SOD and CAT to judge the stress response of corals after infection. KCl and H_2_O_2_ were used to evaluate the efficacy of treatment for ciliate infection. KCl is commonly used as a toxicity test in environmental monitoring; excessive amounts of salts in the environment are toxic and have an LC_50_ that varies for each type of living creature [17,18]. KCl is commonly used in seawater aquarium tanks to prevent flatworm hazards. H_2_O_2_ is currently being considered a potential disinfectant in aquaculture [19]. We hope to find a method that can kill ciliates without harming coral tissues, which will contribute to large-scale coral aquaculture.

## 2. Materials and Methods

### 2.1. Experiment 1: Culture and Drug Therapy of Ciliates

#### 2.1.1. Purification and Identification of Ciliates (*Philaster Lucinda*)

The species were obtained from *G. columna* coral from a TCK coral farm (Pingtung, Taiwan). After the coral tissues were washed with sterilized seawater, ciliates were cultured in 10 mL sterilized seawater tubes. *G. Columna* polyps were collected and fed every day. Twelve hours after feeding, a microscope (Leica DM500 microscope at 400×) was used to observe whether there were zooxanthellae in the bodies of the ciliates. If the zooxanthellae in the body had been digested, the polyps would have been fed. Half of the sterilized seawater was replaced once a week to ensure stable water quality. Macroscopic examination was used to assess the morphology of purified cultured samples [1]. The total DNA of the ciliate samples was extracted using a genomic DNA isolation kit (Protech Technology Enterprise Co., Ltd., Taipei, Taiwan). The universal eukaryotic 18S rRNA gene was amplified with primers 4617f and 4618r [1]. The PCR conditions were as follows: Preheat at 95 °C for 3 min for 35 cycles (95 °C for 30 s, 55 °C for 30 s, and 72 °C for 1 min), then 72 °C for 5 min. Next, 1 uL of the PCR product was used for the nested PCR with the ciliate-specific primers 384f-cil and 1147r-cil [1]; the annealing temperature of the PCR was 60 °C. The PCR product was cloned into pCR-Blunt (Zero Blunt cloning kit; Invitrogen) and sequenced by a biotech company. The sequence was aligned using the Basic Local Alignment Search Tool (BLAST).

#### 2.1.2. Culture of Ciliates

The purified ciliates were cultured in 50 mL test tubes, and the culture container and seawater were sterilized before use. The initial culture density was 1 cell mL, and *G. columna* polyps were fed every day. The cell density was measured at 48 h by direct counting using a microscope at (Leica DM500) at 100× magnification with a hemocytometer during the assay [20]. According to the method outlined in [20], specific growth rates (SGRs) were calculated by hours before and at the end of experiment and cell densities (cells mL^−1^) to determine the growth status of ciliates.

#### 2.1.3. Drug Tolerance Test for Ciliates

Referring to the method of [20], the cultured ciliates were counted using a hemocytometer, and their health status and vitality were observed under a microscope. The ciliate density used in this study was 1 cell mL^–1^. Then, KCl and H_2_O_2_ concentrations of 0.05, 0.1, 0.5, 1, 1.5, and 2% were used to carry out a toxicity test on the ciliates, and the time of death and number of ciliates were recorded until the ciliates had died completely, that is, the experiment ended. The endpoint was mortality, expressed as the LC_50_ value (lethal concentration for 50% of the population).

### 2.2. Experiment 2: Treatment of Coral Ciliate Diseases

#### 2.2.1. Source of Coral Samples

A hundred colonies of *G. columna* were obtained from Coral King Coral Farm (Pingtung, Taiwan), a CITES legal coral farm (CITES number No.FTS507W0153796). The specimens were maintained and fed in an aquarium (60 × 35 × 30 cm^3^) with a recirculating filtered seawater system; refer to Ding et al., 2021 [21] for the coral breeding method. After two months of acclimation and self-repair, the healthy corals were segmented into a colony containing five polyps, and then fixed on porous foundation stones with coral glue, each group containing 10 colonies. Each experiment was repeated three times, each group containing 30 colonies (n = 30 colonies). The water quality parameters of the coral aquaculture are shown in Table 1.

#### 2.2.2. Treatment Trial of Ciliate Infection

The specimens were maintained and fed in an aquarium (60 × 35 × 30 cm^3^) with a recirculating filtered seawater system, using a 500 mL beaker filled with sterilized seawater for the experimental grouping. The beaker was placed in a thermostatic circulating filtration aquarium to ensure that the environment is consistent with the water temperature conditions. In experiment 1, the LC_50_ was known to be 1.50 ppm in the inhibition experiment of drugs on ciliates. Therefore, in the study of the drug tolerance and ciliate inhibition of coral, 1.5 and 2% were selected as test concentrations, denoted by 1.5 and 2. The experiment was divided into ciliate infection (Y) and non-infection (N), ciliate infection without drug treatment as the control group (C), totaling 72 h of treatment. At the end of the experiment, the polyp contraction of coral was measured by the method of [22] to judge the change in coral morphology, and the survival rate, zooxanthellae number, chlorophyll a, SOD, and CAT were measured to evaluate the effect of drug treatment on coral. After the experiment, the seawater in the beaker was centrifuged, the supernatant liquid was removed, and the ciliates in the seawater were calculated by a hemocytometer.

#### 2.2.3. Stress Response of Ciliates to Corals

The *G. columna* was cultured in a 500 mL beaker and infected with 1 cell mL of ciliates. The changes in SOD and CAT in corals were observed for 72 h, and samples were taken every 12 h to investigate the stress of ciliates on corals, with 10 polyps per group. All experiments were performed in triplicate, and thus each group involved a total of 30 colonies. This experiment was conducted to evaluate the changes in SOD and CAT within 72 h of two antioxidant enzymes in corals infected with ciliates.

#### 2.2.4. Antioxidant Enzyme Analysis

##### Preparation of Coral Tissue Solution

This experiment refers to the detection method [23], where tissues were removed from the coral fragments, as described below, and dissolved in nine volumes of ice-cold extraction buffer (20 mM phosphate buffer, 1 mM EDTA, 0.1% (*v/v*) Triton X-100; pH 7.4). The resulting crude extract was sonicated on ice (five times, 3 s each), centrifuged at 12,000× *g* for 5 min at 4 °C, and used as the tissue solution for protein and enzyme assays.

##### Superoxide Dismutase Detection

SOD activity was assayed spectrophotometrically as described by Elstner and Heupel (1976), Oyanagui (1984), and [14]. The standards for activity were prepared using bovine erythrocytic SOD and CAT (Sigma) for each set of samples. Next, 15 mL of the tissue’s solution was placed into the test tube, and then 10 mL of 0.1 mole PBS buffer was added and centrifuged at 1500× *g* for 30 min. The supernatant was taken as the sample. All assays were conducted at 25 °C, and enzyme activity was expressed as units (U) per milligram of protein. The protein content was determined by the Bradford assay [24].

##### Catalase Detection

CAT was determined using a commercially available reagent set (Sigma). For experimental operation reference, Main, Ross, and Bielmyer’s (2010) [25] catalase measurement method for detection. First, sample preparation was carried out. Then, 15 mL of the tissue solution were placed into the test tube, and then 10 mL of 0.1 mole PBS buffer was added and centrifuged at 1500× *g* for 30 min. The supernatant was taken as a sample. The sample was thoroughly mixed with a blank, 25 µL of colorimetric assay substrate solution was added, and the solution was left to stand for 1~5 min. Afterward, 5 µL of the sample was placed into a test tube, 70 µL of 1× assay buffer was added, and then 75 µL of blank 1× assay buffer was added. Next, the sample was thoroughly mixed with the blank, adding 25 µL of colorimetric assay substrate solution before being left to stand for 1~5 min. Following this, 900 µL of stop solution was added to the sample and blank, before being mixed upside down. Then, 10 µL of reaction mixture was moved into another test tube, after which 1 mL of color reagent was added, which was left for 15 min at room temperature before measuring the absorbance value at OD 520 nm.

Calculation formula:

∆A = A_sample_ − A_blank_

CAT (µmoles/min/mL) = (∆A × d × 100)/(V × t)

d: Diluted multiples;

V: The volume of the sample;

t: The reaction time.

##### Protein Concentrations

The *G.* *columna* specimens were sonicated, and protein concentrations were measured using a Bradford protein assay kit (Amresco, Solon, OH, USA), with bovine serum albumin serving as the protein standard.

#### 2.2.5. Analysis of Zooxanthellar Density and Chlorophyll a

At the end of the 72 h experiment, the coral tissues were homogenized, and the number of zooxanthellae in the *G. columna* was observed and calculated with a blood cell counter according to [26]. The zooxanthellae density is expressed as number per polyp. For the determination of the chlorophyll a content, according to the methods of [22,26], the fresh coral tissue (0.5 g) was first homogenized, then 10 mL of 90% acetone was added to extract chlorophyll a, before being left to stand for 24 h at 4 °C under all-black conditions. The absorption spectra were measured at 630 and 664 nm using a Hitachi U-2000 spectrophotometer, and the concentration calculated using the equations of [27]. The chlorophyll a content was measured in the immediately sampled *G. columna* colonies as micrograms of chlorophyll a per gram of the wet weight of colony tissues.

### 2.3. Statistical Analysis

Data were obtained from two independent experiments, and the final results are presented as the mean ± standard deviation (SD). One-way analysis of variance and Duncan’s multiple range test were used to determine the statistical significance for SOD, CAT, survival of *G. columna*, and zooxanthellar density and chlorophyll a content in *G. columna*. A *p*-value of <0.05 was considered significant. All statistical analyses were performed using IBM SPSS statistics 20.

## 3. Results

### 3.1. Experiment 1: Culture and Drug Therapy of Ciliates

#### 3.1.1. Identification and Morphological Observation of Parasites

**Figure 1:**
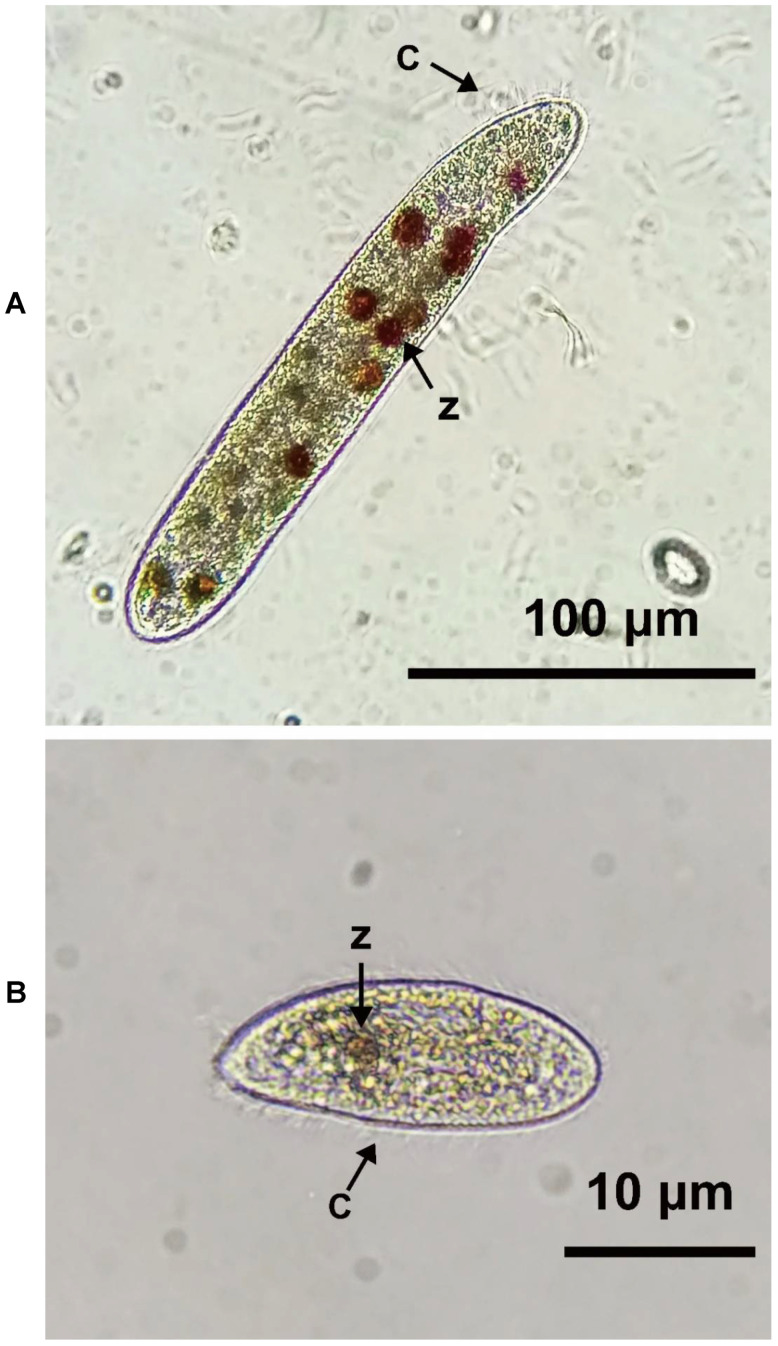
Ciliate morphology (magnification 400×). (**A**): *P. Lucinda*, which was extracted from coral, was in the body full of zooxanthellae with a body length of 170–200 μm and a body width of 30–40 μm. *Cilia* (C), *zooxan thellae* (Z). (**B**): No zooxanthellae were found in the body without feeding, and the body length was approximately 50–70 μm, while the body width was 30–40 μm. Cilia (C), zooxan thellae (Z).

Macroscopic examination allowed a morphological comparison of the purified cultured ciliates, as shown in Figure 1. The body length of this ciliate was long and slender, and its beak is pointed. The body length was approximately 20 μm before feeding. After feeding, it was observed that the body was filled with a large number of zooxanthellae, which made the body 10-times larger. The activity speed was fast, spiralling forward to swim. The color varied from tawny to brown, mainly due to the pigment produced by the endosymbionts being digested. The cilia were ca. 5–10 μm in length, with oral cilia ca. 10–15 μm in length and caudal cilium 12–15 μm in length. The paroral membrane was L-shaped, located on the right-hand side of oral cavity. The length of the buccal field was large, approximately 40–50% of the body. The cytostome was conspicuous and deeply sunk. The macronucleus was band-like, twisted and positioned centrally with several micronuclei attached to it. One small terminally located contractile vacuole was present and often visible. The locomotion on ciliates was characterized by fast, spiral swimming, while rotating irregularly about its main body axis, motionless for short periods when feeding, often seen at the lesion interface or burrowing underneath the tissue and erupting from the mouth of individual polyps [1]. The sequence of cloning was aligned using BLAST and submitted to GenBank (accession number OK030521), and the clones sequenced were affiliated with the 18S rRNA gene of ciliates from Acropora (99% sequence identity). According to the observation of DNA and morphology, the ciliate purified and cultured by us was *P. Lucinda*.

#### 3.1.2. Treatment of Ciliates by KCl and H_2_O_2_

According to Table 2, KCl and H_2_O_2_ did not cause the death of ciliates at 0–0.5%, but ciliates could be observed to swim fast under the influence of drugs and began to die after 90 s at 1%. According to the results, the semi-lethal concentration of LC_50_ is approximately 1.54% for KCl and 1.40% for H_2_O_2_, which can cause ciliates to die after 90 s of treatment. Therefore, 1.5–2% of KCl and H_2_O_2_ were used to evaluate the treatment of coral ciliates.

### 3.2. Experiment 2: Treatment of Coral Ciliate Diseases

#### 3.2.1. Effects of Ciliate Infection on Coral

**Figure 2:**
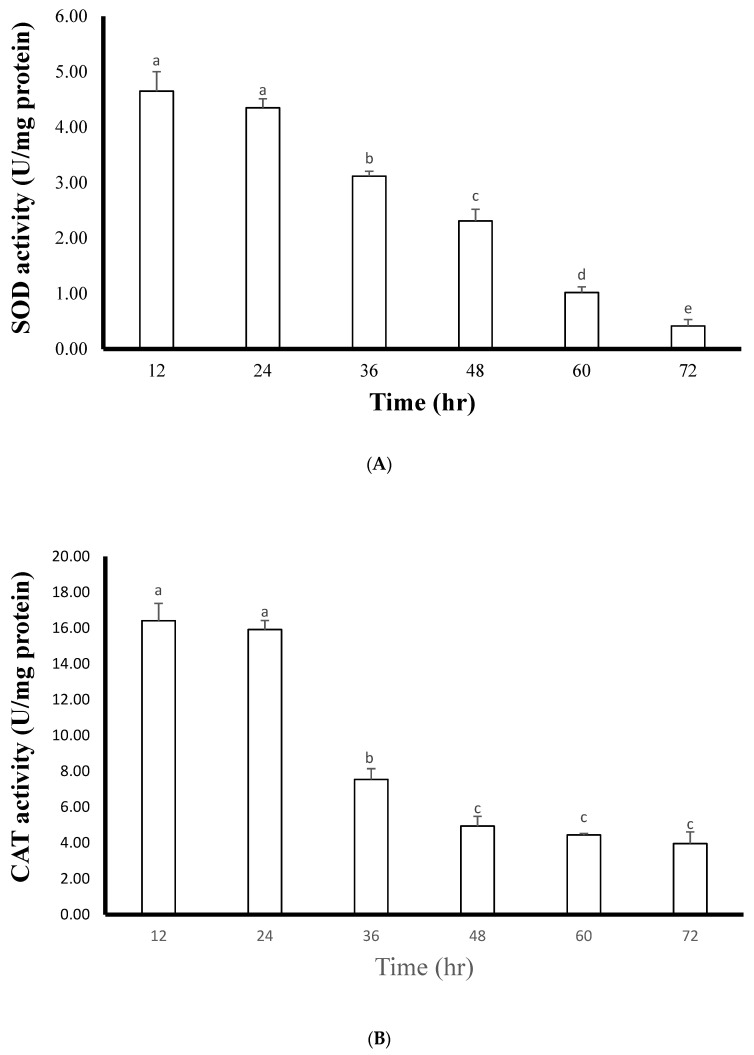
Effects of the SOD and CAT activities on coral infected with ciliate within 72 h. Different letters indicate significant differences among groups (*p* < 0.05). The values are expressed as means ± SDs (n = 30 colonies). (**A**): indicates SOD, (**B**): indicates CAT.

After 72 h of infection, some coral tissues ulcerated and died. A brown gelatinous band layer was produced on the surface of the dead coral. A large number of ciliates could be found in the coral tissues by observing this gelatinous band under a microscope. The SOD and CAT activities were detected to be 4.65 ± 0.35 U/mg of protein and 16.41 ± 0.97 U/mg of protein, respectively, 12 h after infection. After 36 h, the SOD and CAT activities were significantly decreased. The SOD and CAT activities decreased to 0.42 ± 0.12 U/mg of protein and 3.96 ± 0.65 U/mg of protein at 72 h, respectively, which were 11.07 and 4.14 times lower than those at 12 h (Figure 2). The results show that ciliate infection induces a considerable stress response in corals, leading to an increase in ROS levels. In conclusion, the SOD value in coral decreased 24 h after parasite infection, which may have been caused by the rapid proliferation of *P. lucinda* after coral infection, while CAT decreased significantly 36 h after infection. The length of the polyps of infected ciliates shrank 64.33 times after 72 h, and the uninfected polyps could fully extend to 1.93 ± 0.12 cm. Therefore, special attention should be paid to ciliate infection if polyp atrophy is found in large-scale coral aquacultures.

#### 3.2.2. Evaluation of Drug Therapy

By observing the morphological changes in corals after infection, it was found that after 72 h = of *G. columna* infection with *P. lucinda*, the length of the polyps of corals decreased significantly by 64.33 times compared to the C group. Compared to the C group, the length of the polyps in the treatment groups treated with KCl decreased only 1.97 times, while that in the H_2_O_2_ treatment group decreased 19.30 times compared with the C group (Table 3). The number of zooxanthellae in the C group was 0.21 ± 0.17 cells × 10^7^ m^−2^ and the chlorophyll a content was 1.53 ± 0.11 µg cm^–2^. The number (N) of zooxanthellae in the untreated group was 5.23 ± 0.35 cells × 10^7^ m^−2^ and the chlorophyll a content was 58.00 ± 5.29 µg cm^–2^, which were significantly decreased in the control group (Table 3). There were significant differences in the zooxanthellae and chlorophyll a content compared to the C group in each treatment group using 1.5 and 2% KCl (*p* < 0.05). However, the number of zooxanthellae treated with 1.5 and 2% of H_2_O_2_ decreased by 1.79 and 2.00 times, and the number of uninfected zooxanthellae decreased by 1.49 times and 5.03 times, respectively, compared to that of the KCl-infected groups. Treatment of ciliate infection with H_2_O_2_ resulted in a decrease in *G. columna* zooxanthellae and chlorophyll a. The survival rate of C was only 13.33 ± 5.77%, and the survival rate of the other treatment groups was 100% (Table 3).

#### 3.2.3. To Treat Stress Responses to Coral

**Figure 3:**
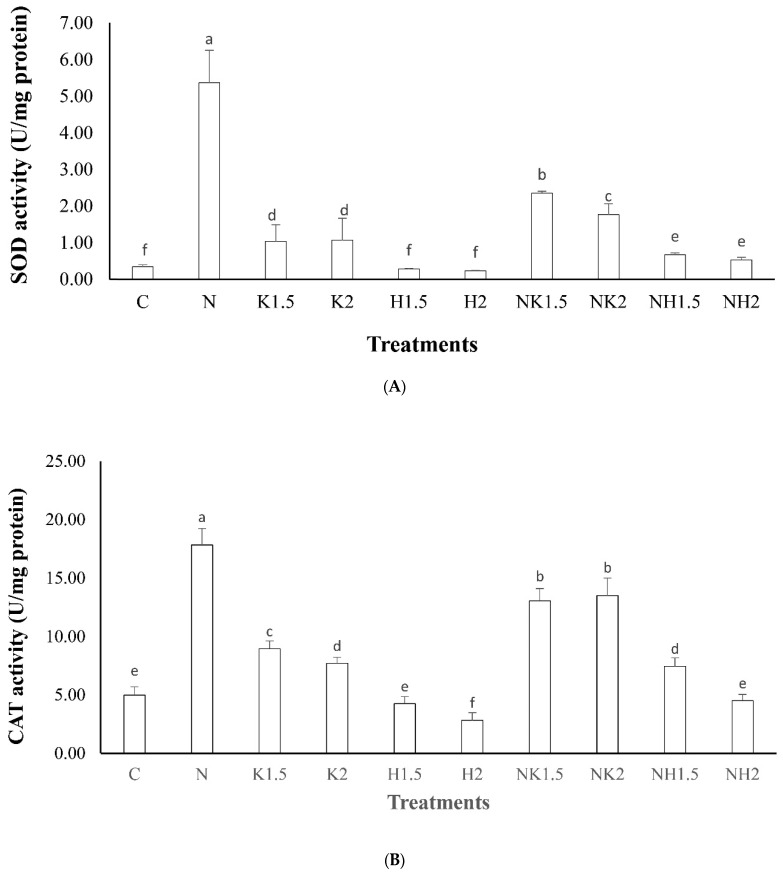
Effects of KCl and H_2_O_2_ on the SOD and CAT activities of coral. Different letters indicate significant differences among groups (*p* < 0.05). Values are expressed as means ± SDs (n = 30 colonies). (**A**): indicates SOD, (**B**): indicates CAT.

As shown in Figure 3, SOD and CAT in all treatment groups were lower than those in the N group, indicating that the dose of drug treatment may lead to the production of a large number of ROS in corals. Prolonged treatment for 72 h may lead to the inability of SOD to resist oxidation stress, resulting in a decline in SOD, the rise of ROS and hydroxyl radical (•OH) in corals, and cause cell damage. The lowest SOD values were C, H 1.5, and H2, which might have been caused by ciliate infection and H_2_O_2_ stimulation, leading to a decrease in the SOD value in coral. In addition, according to the results, the SOD and CAT activities of NH 1.5 and NH 2 treated only with drugs were 3.51 and 3.33 times lower than those of NK 1.5 and NK 2, respectively, which may have been because H_2_O_2_ has a greater impact on the stress response of corals.

## 4. Discussion

At present, there are few research works on coral diseases and medication, so the most serious concerns are such diseases and their treatments in coral aquacultures. If the problem of coral ciliate infection can be solved, it will be of great help to large-scale coral cultivation. According to the observation of DNA and morphology, the ciliate we purified and cultured was *P. lucinda*. This ciliate 100% matched the DNA sequence of *P. lucinda* in GenBank found in the Caribbean [1]. We found that *G. columna*, *E. glabrescens*, *B. violacea*, *Acropora formosa*, *Lemnalia fiava*, etc., are parasitified by *Philaster lucinda* in the TCK coral farm. This causes the ulceration of coral tissue and produces yellow or brown jellylike tissue. Therefore, it was preliminarily determined that it is not an obligate parasite; this is a massive parasitic disease that affects wild or artificial coral aquaculture.

The study of [1] found that *P. lucinda* causes coral death within 24 h after infection. Previous studies have found that survey on ciliate infection of *Acropora muricata* colonies on the great barrier reef found that the tissue decay rate of ciliate infected coral was ~2 mm/day [8]. The tissue decay rate of corals infected with WBD was ~0.8 mm per day [6]. In addition, ciliates have been found to infect corals in the Caribbean, and the rate of tissue decay is around 0.7 mm per day [6]. We observed that ciliates need to eat a large number of zooxanthellae to reproduce in the early stage of parasitism, and the division and reproduction in corals lead to the swallowing of coral tissues and zooxanthellae, which leads to ulceration. When the density of ciliates is too high or food is insufficient, proliferation is inhibited. According to a previous study [1], a large number of zooxanthellae were found in ciliates, which was also observed in this study. Therefore, after infection, zooxanthellae and chlorophyll a in corals decrease, which may be affected by ciliate feeding. However, no damage to the ectoderm tissue was observed during the initial infection of corals. It was concluded that ciliates may invade through the mouth of corals, and previous observations also found that ciliates enter and exit via this route [1]. Therefore, *P. lucinda*, which was not easy to observe from the appearance at the initial stage, had already begun to fester or die when it was discovered. Therefore, it is possible to estimate the health status of corals by macroscopic examination of polyps. Polyps stretch out when healthy and shrink when stressed. The length of uninfected N polyps was 1.93 ± 0.12 cm/polyp, while infected C polyps shrank to only 0.03 ± 0.06 cm/polyp. Previous studies have shown that corals respond to light, water, and food by stretching and contracting polyps [22,28]. Therefore, early infection can lead to the condition of coral polyp atrophy. It can be used as a macroscopic examination of coral disease infection in large-scale coral aquacultures. In addition, A variety of ciliate infection diseases found so far will cause the coral damaged tissues to produce black or jelly like tissue morphology changes [8]. In addition to ciliates, it is also possible to be infected by ciliates and bacterial complex. Previous studies have found that Syndrome (WS) and Brown band disease (BrB) is an important disease-causing coral death. Four bacterial and nine ciliate ribotypes were observed in both diseases. However, there is little research on coral diseases at present. In our study, it was found that infection by ciliates would lead to coral ulceration and death [29].

After 72 h of ciliate infection, the SOD and CAT activities of corals decreased by 11.07 and 4.14 times, respectively, and the affected corals suffered a considerable stress response, leading to an increase in ROS in vivo. Previous studies have found that disease, environment, temperature, and light can all cause stress responses in corals [11], although coral can inhibit ROS and prevent cell damage [10]. During the early stage of ciliate infection, when the cells are not seriously damaged, a large number of antioxidant enzymes are produced to inhibit ROS. However, after a large number of ciliates proliferate in coral tissues, these tissues become seriously damaged, resulting in a decline in SOD and CAT, finally resulting in coral death.

The results showed that the treatment of 1.5% of KCl had a good inhibitory effect on ciliate infection, which could kill ciliate in a short time and improve the survival rate of corals without causing polyp atrophy. Potassium chloride (KCl) is commonly used in food as a substitute for NaCl salt [30]. It can be used to regulate the osmotic pressure and kill ciliates. Moreover, 1.5% of KCl in *G. columna* is acceptable and does not cause coral polyps to atrophy or die. A previous study [31] found that KCl can inhibit the growth of *Listeria monocytogenes*. In addition to this, KCl for *Aeromonas hydrophila*, *Enterobacter sakazakii*, *Shigella flexneri*, *Yersinia enterocolitica*, and *Staphylococcus aureus* also have bacteriostatic functions [30].

In the treatment of ciliate infection with KCl and H_2_O_2_, it was found that KCl treatment had little effect on the number of *G. columna* SOD, CAT, and zooxanthellae, and H_2_O_2_ treatment led to a decrease in coral SOD and CAT, as well as a decrease in the zooxanthellae content, resulting in the occurrence of polyp contraction. This may have been caused by the stress response of H_2_O_2_-induced ROS production by corals and zooxanthellae [14]. The authors of [14] showed that when the concentration of H_2_O_2_ in the environment increased to 3 μM, the CAT activity increased, while the SOD activity was not affected. When the temperature increased by 31 °C, the SOD activity decreased but the CAT activity increased. Therefore, when coral is parasitized by ciliates, the activities of SOD and CAT, two antioxidant enzymes, increase. However, with the increase in parasitism time, coral tissues become severely damaged, resulting in a decrease in SOD and CAT activities. Therefore, in the evaluation of drug therapy, SOD and CAT activities can be used to judge the oxidative damage of coral tissues. According to the results, in the evaluation of drug therapy for large-scale coral aquacultures, 1.5% of KCl is recommended to be used for drug bath treatment, which can effectively treat ciliate-infected *G. columna*.

## 5. Conclusions

The results showed that SOD and CAT activities increased after coral was infected by ciliates but decreased with the extension of infection time. Ciliate infection of coral can cause ulceration or death in a short time, causing serious damage to coral. The observation of polyp morphology and antioxidant enzyme activity showed that ciliate infection caused a strong stress response in a short period of time, and urgent drug treatment was necessary to avoid coral casualties. KCl can be used in the treatment of ciliate infection. In addition to the treatment of ciliate infection, KCl can also be used for the quarantine of corals before they are put into aquarium tanks to achieve disease prevention. The recommended concentration for *G. columna* treatment is 1.5%, which can effectively poison ciliates and restore coral polyps to their full form in a short time without causing a coral stress reaction. This research has been applied in TCK coral farm, effectively preventing some corals from being infected by *P. lucinda*. It is hoped that this research can be applied in large-scale aquacultures of corals.

## Figures and Tables

**Table 1:** The water quality conditions in the study.

Water Quality Conditions	
Temperature	25.5 ± 0.5 °C
pH	8.0 ± 0.5
Dissolved oxygen	5.00 ± 0.05 ppm
Nitrous acid	0.01 ± 0.05 ppm
Nitric acid	0.05 ± 0.05 ppm
Calcium	425 ± 30.12 ppm
Magnesium	1345 ± 40.25 ppm
Ammonia nitrogen	0.01 ± 0.05 ppm
Phosphate	0.01 ± 0.01 ppm

Values are expressed as means ± SDs (n = 3).

**Table 2:** Inhibitory concentrations of KCl and H_2_O_2_ on ciliates.

		Survival Rate% (Mean ± SD)						
	Sec	C	0.05%	0.1%	0.5%	1%	1.5%	2%
KCl	30	100 ± 0.00	100 ± 0.00	100 ± 0.00	100 ± 0.00	82.67 ± 8.33	41.73 ± 5.60	13.60 ± 2.12
	60	100 ± 0.00	100 ± 0.00	100 ± 0.00	100 ± 0.00	46.67 ± 6.11	40.00 ± 8.00	6.80 ± 1.74
	90	100 ± 0.00	100 ± 0.00	100 ± 0.00	100 ± 0.00	36.00 ± 4.00	13.33 ± 6.11	0.00 ± 0.00
H_2_O_2_	30	100 ± 0.00	100 ± 0.00	100 ± 0.00	100 ± 0.00	85.33 ± 10.07	41.73 ± 5.60	10.67 ± 3.59
	60	100 ± 0.00	100 ± 0.00	100 ± 0.00	100 ± 0.00	42.53 ± 4.46	38.00 ± 3.02	7.20 ± 3.17
	90	100 ± 0.00	100 ± 0.00	100 ± 0.00	100 ± 0.00	35.73 ± 3.61	13.47 ± 3.06	0.00 ± 0.00

Values are expressed as means ± SDs (n = 3).

**Table 3:** Effects of KCl and H_2_O_2_ on zooxanthellae, chlorophyll a, and the survival rate of corals.

	Treatments (%)	Zooxanthellae (Cells×10^7^ m^−2^)	Chlorophyll a (µg cm^−2^)	Polyps Length (cm/polyps)	Survival Rate (%)
**Ciliate Tttack**	**C** **K1.5** **K2.0** **H1.5** **H2.0**	0.21 ± 0.17 ^c^ 4.83 ± 0.50 ^a^ 4.93 ± 0.38 ^a^ 2.70 ± 0.46 ^c^ 2.47 ± 1.36 ^c^	1.53 ± 0.11 ^c^ 48.33 ± 5.03 ^a^ 52.33 ± 3.51 ^a^ 33.00 ± 8.72 ^b^ 36.67 ± 7.23 ^b^	0.03 ± 0.06 ^d^ 1.00 ± 0.20 ^b^ 0.83 ± 0.21 ^b^ 0.20 ± 0.21 ^c^ 0.10 ± 0.00 ^d^	13.33 ± 5.77 ^b^ 100 ± 0.00 ^a^ 100 ± 0.00 ^a^ 100 ± 0.00 ^a^ 100 ± 0.00 ^a^

**Y**					
**N**	**K1.5**	4.43 ± 0.40 ^a^	50.67 ± 1.53 ^a^	1.07 ± 0.12 ^b^	100 ± 0.00 ^a^
	**K2.0**	5.03 ± 0.40 ^a^	53.33 ± 3.21 ^a^	1.20 ± 0.10 ^b^	100 ± 0.00 ^a^
	**H1.5**	2.97 ± 0.81 ^b^	29.00 ± 4.36 ^b^	0.07 ± 0.06 ^d^	100 ± 0.00 ^a^
	**H2.0**	2.40 ± 1.04 ^b^	24.67 ± 4.04 ^b^	0.03 ± 0.06 ^d^	100 ± 0.00 ^a^
	**N**	5.23 ± 0.35 ^a^	58.00 ± 5.29 ^a^	1.93 ± 0.12 ^a^	100± 0.00 ^a^

C: No processing; N: *P. lucinda* infection not using drugs; K: KCl; H: H_2_O_2_; Y: Infected with *P. lucinda*; N: Not infected with *P. lucinda*. Different letters indicate significant differences among groups (*p* < 0.05). Values are expressed as means ± SDs (n = 30 colonies).

## Data Availability

Not applicable.
